# *Rickettsia rickettsii* inactivated whole cell antigen vaccine protects against Rocky Mountain spotted fever independent of the adjuvant used

**DOI:** 10.1128/iai.00412-25

**Published:** 2025-10-29

**Authors:** Perle Latré de Laté, Ian M. Stoll, Jonathan Ferm, Swetha Madesh, Dominica Ferm, Deepika Chauhan, Debika Choudhury, Huitao Liu, Anish Yadav, Suhasini Ganta, Dae Y. Kim, Jodi L. McGill, Roman R. Ganta

**Affiliations:** 1 Department of Pathobiology and Integrative Biomedical Sciences, College of Veterinary Medicine, University of Missouri 219018, Columbia, Missouri, USA; 2 Veterinary Medical Diagnostic Laboratory, Department of Pathobiology and Integrative Biomedical Sciences, College of Veterinary Medicine, University of Missouri 365852, Columbia, Missouri, USA; 3 Department of Veterinary Microbiology and Preventive Medicine, College of Veterinary Medicine, Iowa State University 537366 , Ames, Iowa, USA; University of California Davis, Davis, California, USA

**Keywords:** RMSF, *Rickettsia*, Rocky Mountain spotted fever, tick-borne pathogens, vaccines, vector-borne diseases

## Abstract

Rocky Mountain spotted fever (RMSF) caused by *Rickettsia rickettsii* is the most fatal tick-borne disease in people and dogs in the Americas. This pathogen is transmitted by several hard ticks: *Dermacentor* species, *Rhipicephalus sanguineus*, and *Amblyomma americanum*. RMSF can quickly progress to a life-threatening illness with fatalities ranging from 30% to 80%. Doxycycline is the only treatment option, and currently, no methods are available to prevent RMSF. We previously reported that vaccination with *R. rickettsii* whole cell antigen vaccine (WCAV) with Montanide gel adjuvant offers protection against virulent *R. rickettsii* infection in dogs. Here, we compared three adjuvants to optimize the safety and immunogenicity of WCAV: Alhydrogel, Montanide, and Quil A. Independent of adjuvants, all vaccinees were protected, whereas unvaccinated dogs developed the clinical disease. Vaccination reduced the pathogen to undetectable levels in blood and various tissues. An *R. rickettsii-*specific IgG response was observed following primary vaccination in all vaccinated groups, which augmented after booster. The vaccine with Quil A had the highest IgG response with a significant rise in CD4^+^ and CD8^+^ T cell numbers, while Montanide and Alhydrogel resulted in a balanced IgG response. IgG2 was the primary antibody detected in unvaccinated infection controls. Several systemic proinflammatory cytokines varied after infection in both vaccinees and controls. Plasma concentration of intercellular adhesion molecule 1 was higher in unvaccinated compared to vaccinees in the first 9 days after infection. This study demonstrates that the WCAV efficacy is independent of the adjuvant, although Quil A induced a higher IgG response and expansion of CD4 and CD8 T cells.

## INTRODUCTION

Rocky Mountain spotted fever (RMSF) is a potentially fatal tick-borne disease impacting dogs and people. RMSF is frequently reported in several countries of North, Central, and South America, including in the United States. A high incidence of fatal RMSF cases was also reported in Mexico in recent years (1–4). The causative agent *Rickettsia rickettsii*, an obligate, intracellular bacterial pathogen, is transmitted by several hard ticks, including *Dermacentor* species, *Rhipicephalus sanguineus*, and *Amblyomma americanum* (5–11). *Dermacentor variabilis* and *Dermacentor andersoni* are regarded as the major vectors of *R. rickettsii* in the United States (8, 9), although many documented cases on Indian reservations of Arizona and New Mexico are linked to *Rhipicephalus sanguineus* (5, 6, 10, 11). *Amblyomma americanum* and *R. sanguineus* are the primary vectors of *R. rickettsii* in Mexico and South American countries (5, 9, 11). Early clinical signs in dogs and people occur in less than a week following a tick bite. They include elevated body temperature, headache, nausea, muscle pain, stomach pain, lack of appetite, and petechial rashes initially appearing on the wrists and ankles, which gradually spread to other parts of the body (1, 12–16). RMSF is characterized by proinflammatory and procoagulant changes, the development of systemic vasculitis, and vascular damage that can affect various organs, including the brain, lungs, liver, skeletal muscles, kidneys, and skin (17–22). Doxycycline is the recommended treatment and is effective when orally administered during the early stages of infection (1, 23–25). If treatment is delayed or untreated, the disease quickly progresses to life-threatening illness, resulting in high mortalities, particularly in young children (ages 0–11 years) and similarly in young dogs (ages 0–6 months) (1, 26–28). In recent years, many fatal RMSF cases ranging from 30% to 80% have been reported in Mexico (26, 29–33) and parts of the United States (7, 8). Currently, there is no vaccine to prevent RMSF, and the disease diagnosis remains complex due to extensive clinical manifestations, which are often mistaken for symptoms of viral and other bacterial diseases (28, 34–36). Hence, there is a critical need to find an efficacious vaccine and new therapies. Several studies reported that the bacterial antigens involved in the stimulation of immunity are likely to yield protective responses against infection (37–41). The major surface proteins of *R. rickettsii,* outer membrane proteins A and B (OmpA and Omp B, respectively), are considered important antigens due to their ability to stimulate an immune response against RMSF in the murine host (37, 38). Subunit vaccine assessments using OmpA, OmpB, and Adr2 (another immunogenic surface protein) of *R. rickettsii* suggest that these proteins stimulate B cell and Th1-type T cell responses and offer protection in the guinea pig and mouse models (40, 42). In our previous study, we reproduced fatal RMSF disease in the physiologically relevant canine host (43). We also reported that a recombinant vaccine containing Adr2 and OmpB-4 does not offer protection in the canine host, while the whole organism-derived inactivated antigen vaccine (WCAV) serves as an ideal candidate vaccine, providing complete protection against a severe form of RMSF (43).

In the current study, we compared the WCAV immune protection using three different adjuvants: aluminum hydroxide (Alhydrogel), Montanide, and Quil A, in support of optimizing the safety and immunogenicity of WCAV using the canine infection model. Our findings confirm that independent of the adjuvant used, all WCAV-vaccinated dogs were protected from developing clinical disease, bacteremia, and tissue pathology. While the vaccine with Quil A adjuvant induced significantly greater antigen-specific IgG1 and IgG2 responses, as well as CD4^+^ and CD8^+^ T cell expansion, we observed no notable differences in protective immunity for the three adjuvants assessed. This study will pave the way for a better understanding of the host adaptive immune response and continuing efforts toward the development of RMSF vaccines broadly applicable for use in preventing the disease in dogs and people.

## RESULTS

### Clinical signs are evident primarily in unvaccinated dogs

**Fig 1:**
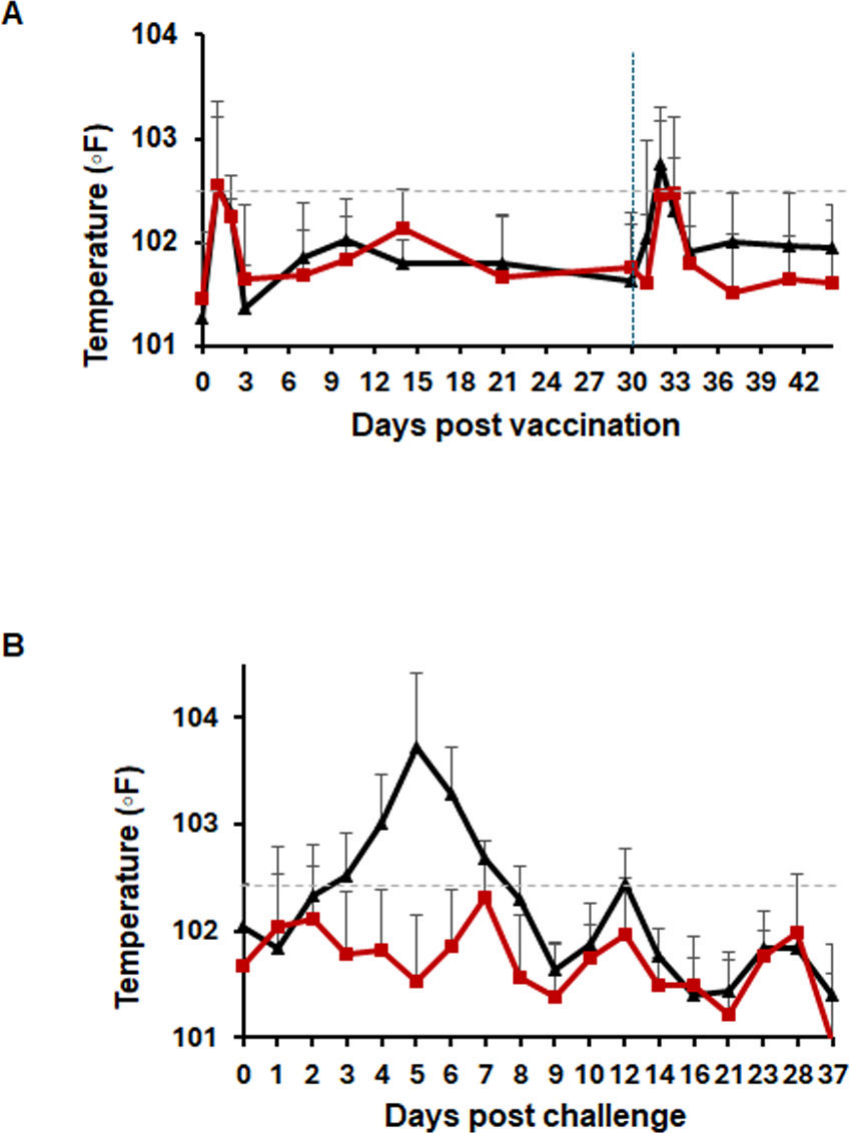
Vaccinated dogs (red line) maintained normal body temperature, while unvaccinated dogs (black line) had fever. Body temperatures measured during the vaccination phase (**A**) and following the infection challenge (**B**). Vertical dashed blue line in panel A indicates the booster vaccine day. The gray dashed-dotted line indicates the fever threshold, above which animals are considered febrile (>102.5°F).

To optimize the safety and immunogenicity of our recently described WCAV in preventing RMSF in the canine infection model (43), we evaluated immune protection with three different adjuvants: Montanide, Quil A, and Alhydrogel. Three groups of dogs were vaccinated with the WCAV prepared using culture-derived *R. rickettsii* Sheila Smith strain, with each group receiving the vaccine with a different adjuvant. Vaccinated dogs received primary and booster vaccinations, while unvaccinated dogs received only adjuvants. All dogs in vaccinated and unvaccinated groups received an intravenous (IV) infection with the *R. rickettsii* Sheila Smith strain. All dogs developed mild inflammation at the injection sites following the vaccination, which was cleared within 3–7 days. All dogs in the unvaccinated (black line) and vaccinated groups (red line) maintained normal body temperature prior to the challenge, although there was a brief increase in body temperature in the days following the prime and booster vaccinations (Fig. 1A). No differences in body temperature were observed among the adjuvant treatment groups following the mock vaccinations. Following the infection challenge, only unvaccinated dogs exhibited high fevers during the first week (Fig. 1B), while all vaccinated animals maintained normal body temperatures, independent of the adjuvant used (Fig. S1). Visible clinical symptoms included petechial rashes on the testes and ears of unvaccinated dogs that received the infection. Five out of six dogs in the unvaccinated group developed rashes (83%) by days 7–9, while only 3 of 15 dogs (20%) in the three vaccinated groups had mild rashes, and this result was independent of the adjuvant used (Table 1; Fig. S2). Severe redness on the testes of all male dogs (three of three) was evident for unvaccinated dogs from day 5 onward, while only 25% of vaccinated males remained healthy (two of eight) and had mild redness in the testes, which was independent of the adjuvant used (Table 1; Fig. S2). Three dogs in the control group had signs of lethargy and depression by day 4 post-infection, which continued for several days, while all vaccinated dogs remained bright, alert and responsive.

**TABLE 1:** Dogs showing severe symptoms: petechiae and redness in testes

Symptom	Days post-infection	Affected/total dogs (%)	
		Controls: 3 males and 3 females	Vaccinated: 8 males and 7 females
Petechiae-ecchymosis	5–6	3/6 (50)	1/15 (7)
	7–9	5/6 (83)	3/15 (20)
	12	Normal	1/12 (8)
Redness in testes	3–4	2/3 (67)	0/8 (0)
	5–9	3/3 (100)	2/8 (25)
	12	Normal	1/6 (17)

### Complete blood count analysis revealed distinct infection-associated changes only in unvaccinated dogs

**Fig 2:**
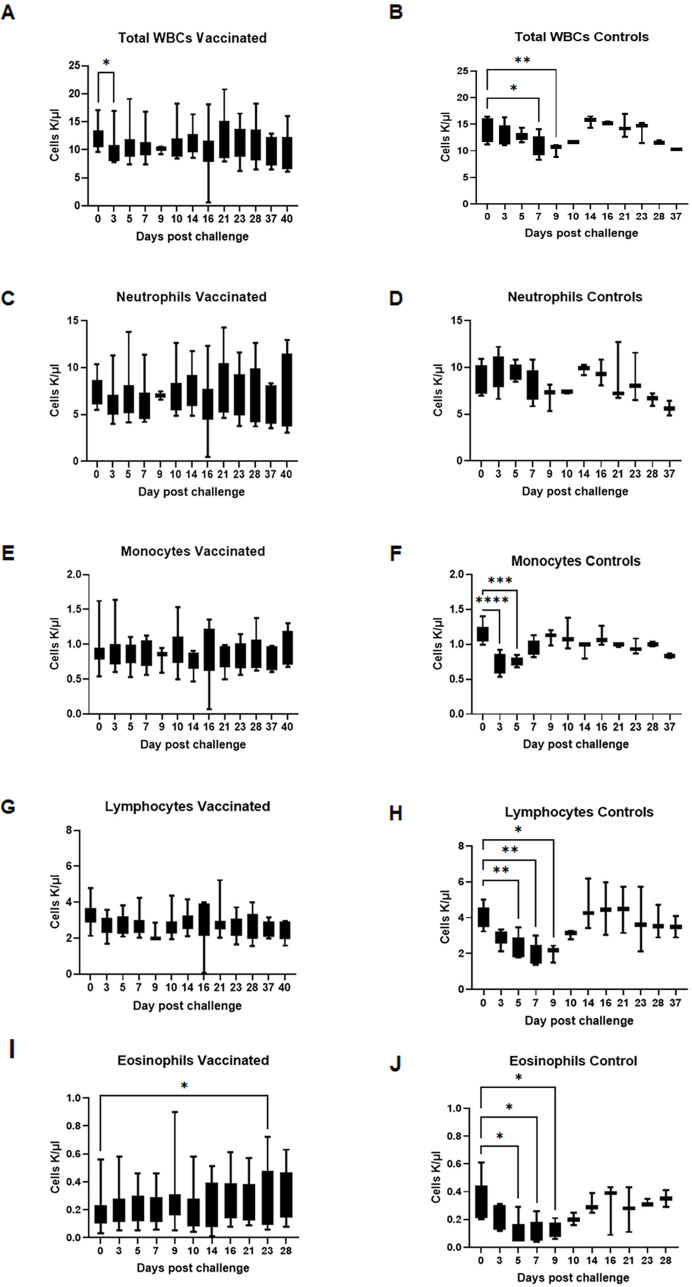
Infection-associated changes in white blood cells were observed primarily in unvaccinated dogs. Complete Blood Count was evaluated in all experimental animals at several time points after vaccination and following infection challenges. Counts of total white blood cells (WCA), neutrophils, monocytes, lymphocytes, and eosinophils were evaluated in vaccinated and control groups. The left panel shows the counts of total white blood cells (WBC) (**A**), neutrophils (**C**), monocytes (**E**), lymphocytes (**G**), and eosinophils (**I**) in vaccinated dogs. The right panels show the data for unvaccinated dogs for WCA (**B**), neutrophils (**D**), monocytes (**F**), lymphocytes (**H**), and eosinophils (**J**). The data were compared by the two-way ANOVA test followed by multiplicity-adjusted *post hoc* comparisons (Dunnett), and a *P*-value < 0.05 was considered statistically significant (*P ≤ 0.05, **P ≤ 0.01, ***P ≤ 0.001, and ****P ≤ 0.0001).

**Fig 3:**
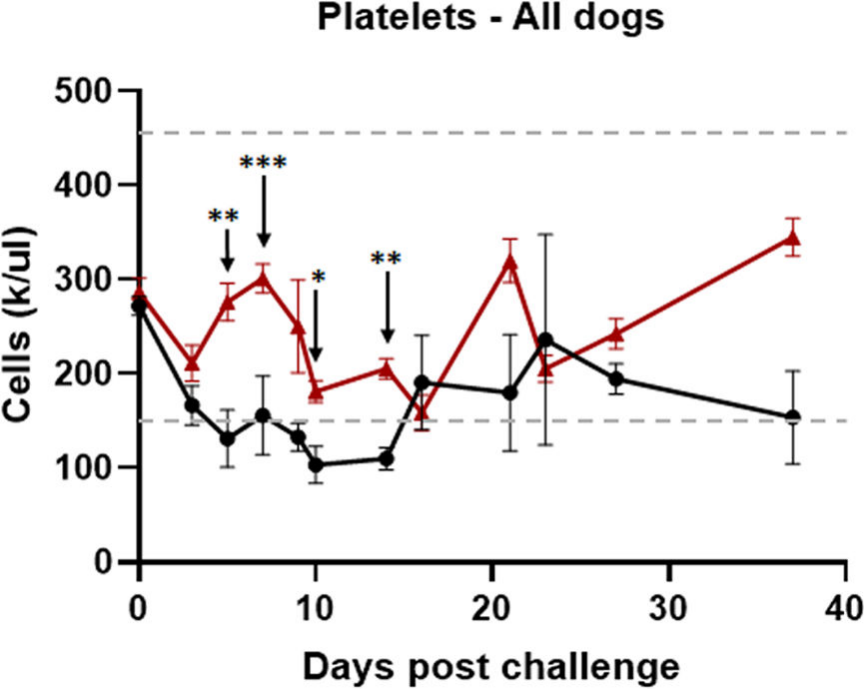
Thrombocytopenia is observed in clinically ill unvaccinated dogs. Platelet decline was significantly different following the infection challenge in unvaccinated dogs (black lines) compared to vaccinated dogs (red lines), which maintained normal platelet values. The horizontal dashed-dotted lines indicate the normal reference range for platelet counts in dogs. The vaccinated and control groups were compared by the two-way ANOVA test followed by multiplicity-adjusted *post hoc* comparisons (Tukey), and a *P*-value < 0.05 was considered statistically significant (*P ≤ 0.05, **P ≤ 0.01, and ***P ≤ 0.001).

Complete blood count (CBC) analysis was performed on several days following vaccination and after infection challenge. Independent of the adjuvant used, all vaccinated dogs maintained normal ranges for the RBC counts, hemoglobin, reticulocyte hemoglobin, and hematocrit levels. Some of the values in five unvaccinated dogs that received *R. rickettsii* infection were lower during the first 2 weeks following the infection challenge (Table 2). In control dogs, total white blood cell (WBC) counts dropped significantly by days 7 and 9 post-challenge and only on day 3 for vaccinated dogs (Fig. 2A and B). Furthermore, division of the specific populations of WBCs showed that while neutrophils had no significant change, they tended to decrease on days 9 and 10 post-challenge in unvaccinated controls, and were unchanged in vaccinated dogs (Fig. 2C and D). Similarly, monocytes were significantly reduced on day 3 post-challenge in unvaccinated controls and remained low through day 5 post-challenge; monocyte levels remained unchanged in vaccinated dogs (Fig. 2E and F). Lymphocyte (Fig. 2G and H) and eosinophil (Fig. 2I and J) levels were significantly reduced on days 5 through 9 post-challenge in unvaccinated controls, while they remained stable in vaccinated dogs. In unvaccinated controls, platelet levels dropped significantly below the normal range, while vaccinated dogs had maintained clinically normal ranges (Fig. 3). Independent of the adjuvants used, all vaccinees maintained normal blood cell profiles for all cell types, although a few differences were evident among vaccinated groups with adjuvant formulation (Fig. S3 and S4). Significant variations were mostly observed in the Alhydrogel vaccinated group (Fig. S3). Otherwise, all different WBCs (Fig. S3) and platelets (Fig. S4) remained within the clinical normal range in each vaccinated group.

**TABLE 2:** Changes in red blood cells, hemoglobin, hematocrit, and reticulocyte-hemoglobin in unvaccinated dogs^*a*^

Animal ID	Days post-infections		
	5	7	9
EFK	Normal	Normal	Normal
GIL2	Normal	Retic-HGB (22.0 g/dL)	
GHL2	Retic-HGB (20.4 g/dL)	Normal	Normal
YBK2	Retic-HGB (20.7 g/dL)	Normal	Normal
IDL2	Retic-HGB (22.0 g/dL) RBC (5.41 M/µL) HGB (12.6 g/dL) HCT (35.0%)	RBC (5.45 M/µL) HGB (12.5 g/dL) HCT (36.2%)	RBC (4.96 M/µL) HGB (11.4 g/dL) HCT (32.8%)
ZXK2	Retic-HGB (21.9 g/dL)	Normal	Normal

^*a*^ Normal values as per Merck veterinary manual: RBC (red blood cell), 5.65–8.87 M/µL; HGB (hemoglobin), 13.1–20.5 g/dL; HCT (hematocrit), 37.3%–61.7%; and retic-HGB (reticulocyte-hemoglobin), 22.3–29.6 pg.

### Vaccinated dogs had reduced systemic bacterial loads

**Fig 4:**
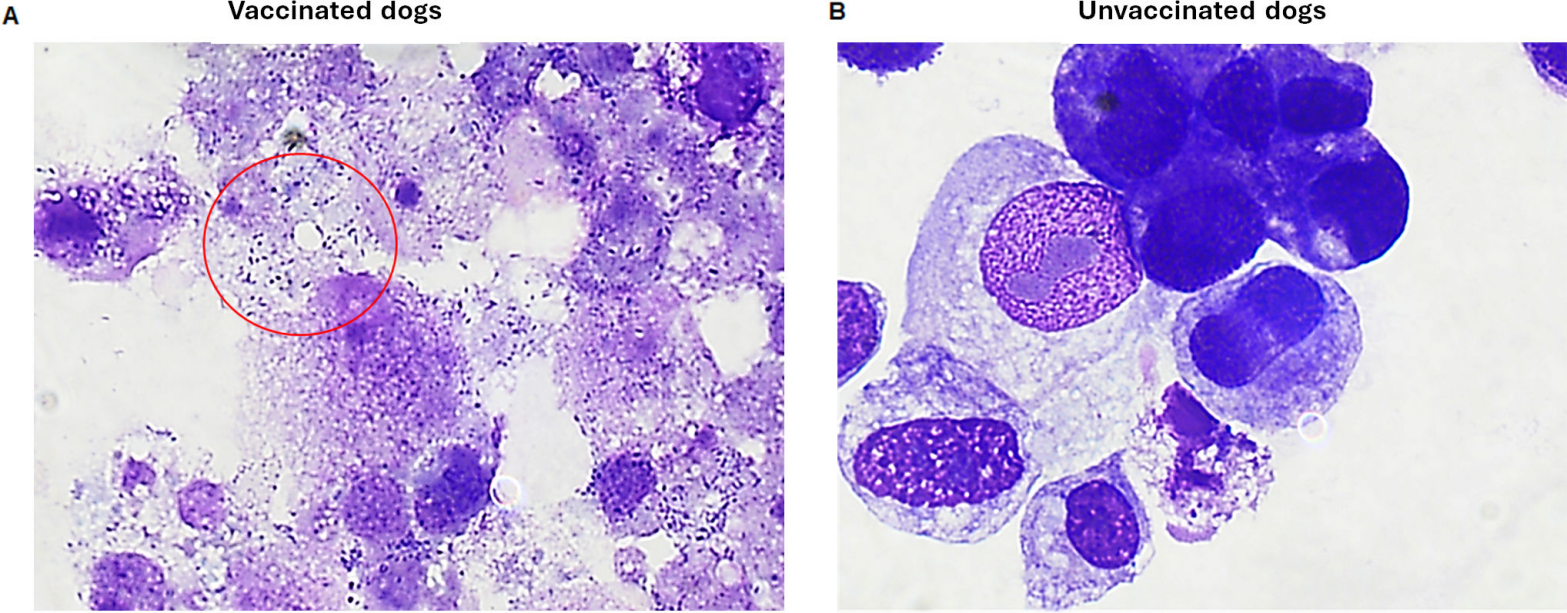
WCA vaccine reduces the pathogen loads to nearly undetected levels as assessed by culture recovery method. Blood of infected dogs collected on several days post-challenge was used to infect Vero cells. The presence of *R. rickettsii* was assessed in culture by microscopy for several days post-inoculation. *R. rickettsii* culture positives were detected only for blood samples assessed from day 7 post-infection challenge from unvaccinated dogs (**A**; as shown with red circle), but not from vaccinated dogs (**B**) that received infection. Red circle highlights bacteria visible as rod-shaped structures (the bacterial infection was independently confirmed by PCR, as discussed in Results).

The presence of systemic *R. rickettsii* was assessed by two independent methods: *in vitro* culture isolation and by nested PCR assay targeting the *adr2* gene (43). Blood samples from several days post-infection challenge were assessed. All six unvaccinated controls tested culture positive for the day 7 post-infection challenge (Table 3; Fig. 4). Independent of the three different adjuvants used, WCAV dogs tested negative for the culture isolation method for all samples assessed (Table 3; Fig. 4). Figure 4 depicts representative microscopy images of blood cultures from control and vaccinated dogs. Similarly, 5 out of 6 dogs in the unvaccinated group tested positive by PCR assay for DNA recovered between days 5 and 9 post-infection, while none of the DNAs from 15 vaccinated dogs tested positive, except for one dog belonging to the Alhydrogel adjuvant group, which tested positive for the DNA recovered on day 10 post-infection (Table 4).

**TABLE 3:** *Rickettsia rickettsii* presence assessed by culture recovery^*a*^

Group of dogs	Adjuvant	Animal	Culture status
Controls	Montanide	EFK2	+
		GIL2	+
	Quil A	GHL2	+
		YBK2	+
	Alhydrogel	ZXK2	+
		IDL2	+
		
Vaccinated	Montanide	GYL2	−
		YGL2	−
		FQK2	−
		GJL2	−
		YKK2	−
		
	Quil A	FSK2	−
		IWL2	−
		YTK2	−
		EOL2	−
		XWK2	−
		
	Alhydrogel	XJK2	−
		ZKL2	−
		EJK2	−
		EGL2	−
		IVL2	−

^*a*^ ^a^Blood samples collected from day 7 post infection from animals were assessed by culture recovery in Vero cell cultures; + and − refer to culture positives and negatives, respectively.

**TABLE 4:** Blood infection status following the infection challenge (Adr2 nested PCR assay)

Animal	Adjuvant	Group	Infection status^*a*^ at indicated days post-infection							
			0	3	5	7	9	10	14	21
Controls	Montanide	EFK2	−	−	−	−	−	−	−	−
		GIL2	−	−	+	−	−	n/a	n/a	n/a
	Quil A	GHL2	−	−	−	+	−	−	−	−
		YBK2	−	−	−	−	+	n/a	n/a	n/a
	Alhydrogel	ZXK2	−	−	−	+	−	−	−	−
		IDL2	−	−	−	+	−	n/a	n/a	n/a
Vaccinated	Montanide	GYL2	−	−	−	−	−	−	−	−
		YGL2	−	−	−	−	−	−	−	−
		FQK2	−	−	−	−	−	−	−	−
		GJL2	−	−	−	−	−	n/a	n/a	n/a
		YKK2	−	−	−	−	−	−	−	−
	Quil A	FSK2	−	−	−	−	−	n/a	n/a	n/a
		IWL2	−	−	−	−	−	−	−	−
		YTK2	−	−	−	−	−	−	−	−
		EOL2	−	−	−	−	−	−	−	−
		XWK2	−	−	−	−	−	−	−	−
	Alhydrogel	XJK2	−	−	−	−	−	−	−	−
		ZKL2	−	−	−	−	−	n/a	n/a	n/a
		EJK2	−	−	−	−	−	−	−	−
		EGL2	−	−	−	−	−	−	−	−
		IVL2	−	−	−	−	−	+	−	−

^*a*^ +, positive; −, negative; n/a, not tested.

### Systemic cytokine responses differ between vaccinated and unvaccinated dogs following infection

**Fig 5:**
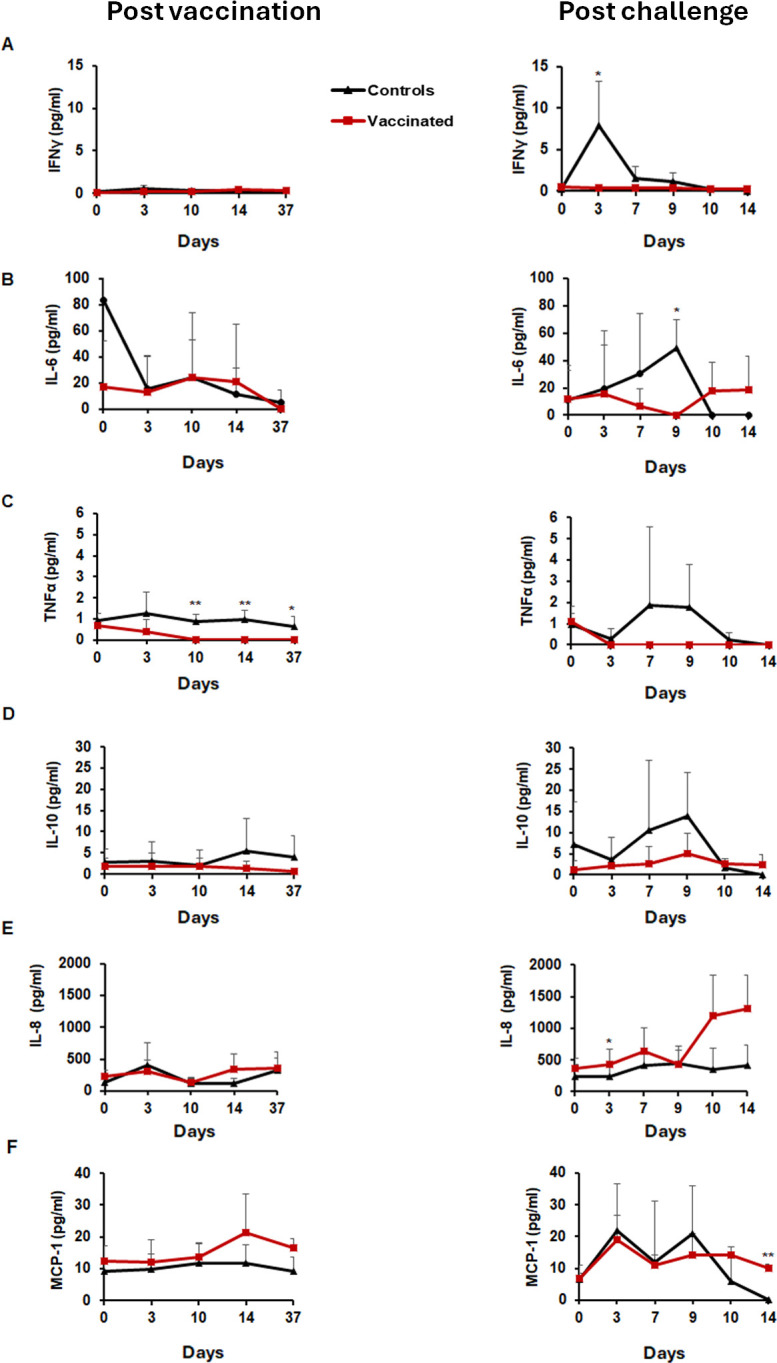
Pro-inflammatory cytokine trends in vaccinated versus unvaccinated dogs after infection. Cytokine and chemokine levels were evaluated for plasma samples collected at different time points by the cytometric bead array. The vaccinated and control groups were compared by the two-way ANOVA test followed by multiplicity-adjusted *post hoc* comparisons (Tukey), and a *P*-value < 0.05 was considered statistically significant (*P ≤ 0.05 and **P ≤ 0.01). Left and right panels (**A–F**) represent the data generated following vaccination or mock vaccination and after infection challenges for the vaccinated and unvaccinated groups, respectively.

Pro-inflammatory cytokines such as IFN-γ, IL-6, and TNF-α are known to be elevated in patients with severe RMSF (44–46). To explore whether vaccination influenced systemic inflammatory responses, we analyzed plasma cytokine levels in vaccinated (*n* = 6) and unvaccinated (*n* = 6) dogs after *R. rickettsii* infection using a Luminex bead-based multiplex assay. Each vaccinated animal had received a WCAV formulation combined with one of three different adjuvants. In vaccinated dogs, plasma levels of IFN-γ remained stable both during the vaccination phase and after infection, whereas a transient but significant increase was observed in unvaccinated controls at day 3 post-infection (Fig. 5A). IL-6 levels were significantly elevated on day 9 post-infection in control dogs compared to vaccinees (Fig. 5B). TNF-α concentrations were elevated in controls during the preinfection stage, on days 10, 14, and 37. After infection, TNF-α was increased from day 3 to day 9 (*P* = 0.003) in control dogs compared to vaccinated animals (Fig. 5C). IL-10 levels did not statistically differ between vaccinated and control dogs (Fig. 5D). Concentrations of IL-8 differed between vaccinated and control dogs only on day 3 post-infection (Fig. 5E). Concentrations of MCP-1 were elevated in vaccinated animals compared to controls on day 14 post-infection (Fig. 5F).

**Fig 6:**
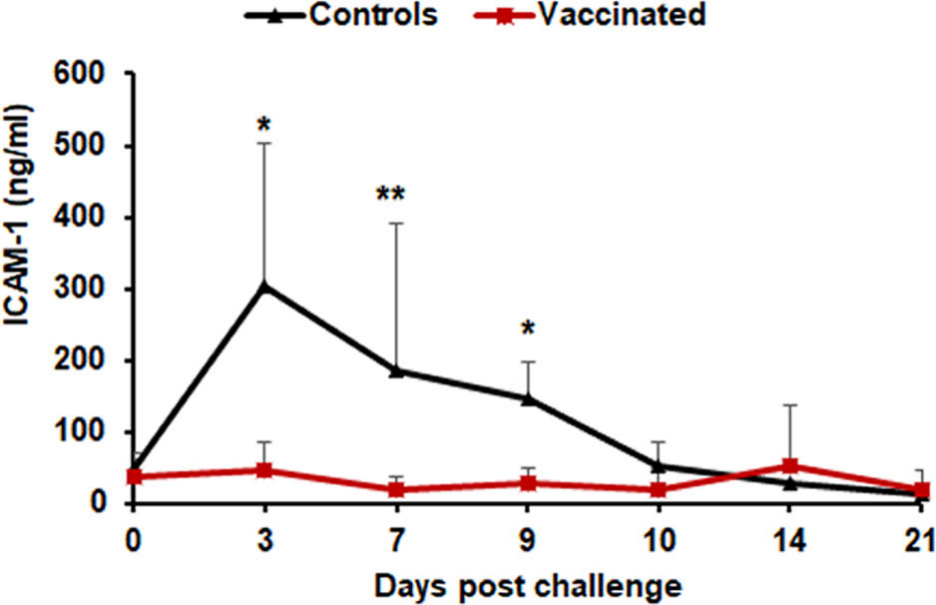
WCAV limits vasculitis. Concentrations of released ICAM-1 and VEGF were evaluated by enzyme-linked immunosorbent assay. Secretion of ICAM-1 remains low in vaccinated animals (red lines), while significantly higher in unvaccinated controls (black lines) from day 3 to day 9 post-infection challenge. The vaccinated and control groups were compared by two-way ANOVA test followed by multiplicity-adjusted *post hoc* comparisons (Tukey), and *P*-value < 0.05 was considered statistically significant (*P ≤ 0.05 and **P ≤ 0.01).

To further assess systemic inflammation and potential vascular involvement, we measured plasma concentrations of intercellular adhesion molecule 1 (ICAM-1), which has been associated with vascular inflammation and vasculitis (47–51). ICAM-1 concentrations were elevated in unvaccinated animals on days 3, 7, and 9 following infection, but did not change in vaccinated dogs (Fig. 6).

### Vaccine-specific humoral response differed for adjuvants used for the WCAV

**Fig 7:**
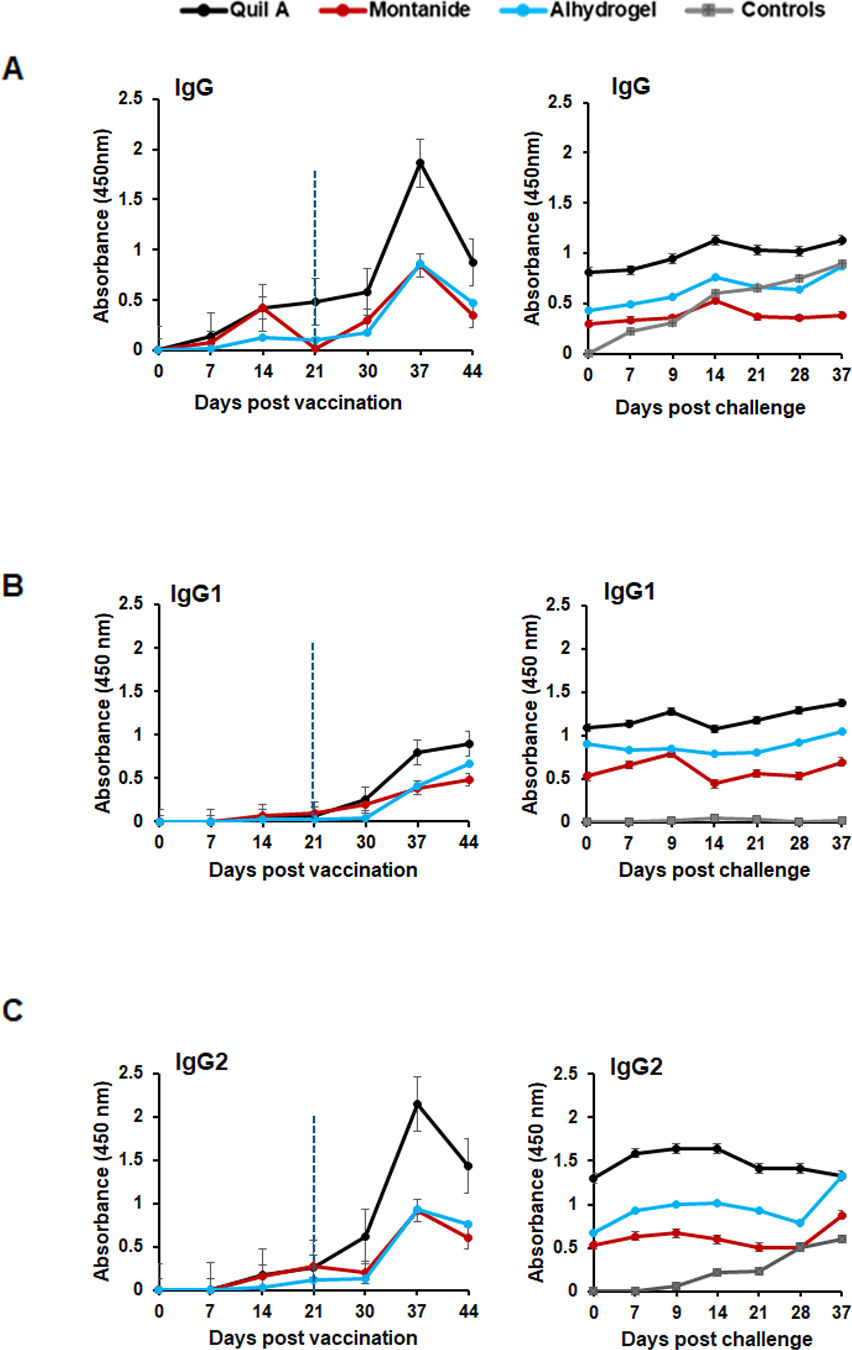
Development of antibodies by vaccinated dogs. Plasma samples collected from all dogs several days following vaccinations and infection challenges were assessed by ELISA for the presence of *R. rickettsii*-specific IgG (**A**), IgG1 (**B**), and IgG2 (**C**). All dogs receiving WCAV developed antigen-specific IgG responses following the primary vaccination, which increased following the booster vaccination. *R. rickettsii*-specific total IgG and IgG subclass; IgG1 and IgG2 in different groups of vaccinated dogs were compared by two-way ANOVA test followed by multiplicity-adjusted *post hoc* comparisons (Tukey). *P*-value < 0.05 was considered statistically significant, which was observed for total IgG and IgG 2 for Quil A compared to Montanide and Alhydrogel from day 37 onward with *P*-values of ≤0.0001. Vertical dotted blue lines refer to the days of booster vaccination.

Plasma samples were collected from all dogs, and *R. rickettsii*-specific IgG responses were assessed by enzyme-linked immunosorbent assay (ELISA). All vaccinated dogs had an increase in *R. rickettsii-*specific IgG, which was detected from day 7 post-vaccination when Quil A was used as the adjuvant, and from day 14 for the Montanide and Alhydrogel adjuvant groups. The response was enhanced following booster vaccination but not after infection challenge (Fig. 7A). The IgG response was substantially higher (more than two times) in dogs receiving WCAV with Quil A compared to Montanide and Alhydrogel. Control dogs were negative for *R. rickettsii-*specific IgG prior to infection but developed a response after infection challenge. IgG subclass evaluation revealed the presence of both IgG1 and IgG2 (Fig. 7B and C) in all three vaccinated groups, with significantly higher IgG2 concentrations observed when Quil A was the adjuvant (*P* ≤ 0.0001). The spike in the IgG2 response with this adjuvant occurred immediately following booster vaccination (Fig. 7C). The IgG responses in unvaccinated dogs following infection challenge were lower and primarily represented as IgG2 (Fig. 7).

### All three vaccinated group dogs developed cell-mediated immune responses

**Fig 8:**
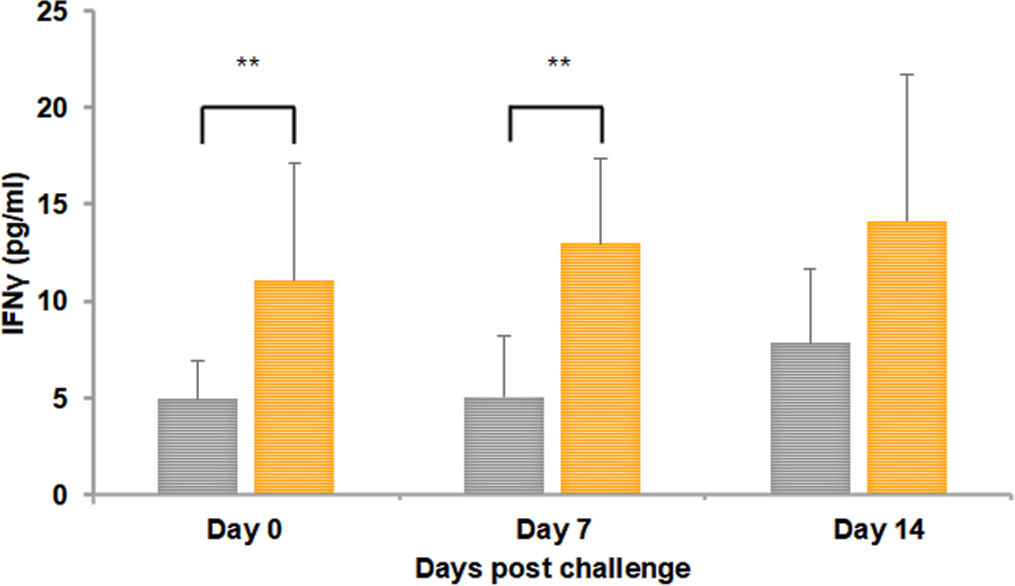
Antigen-specific IFN-γ production by PBMCs. Blood collected from all animals on days 0 and 30 post-vaccination and on days 50, 57, and 64, which are days 0, 7, and 14 post-infection challenges (PC), was assessed for PBMC stimulation and cytokine analysis. PBMCs isolated were stimulated for 5 days with 10 µg/mL whole-cell lysate from *R. rickettsii*. The cell culture supernatants recovered following 5 days in culture were analyzed for IFN-γ expression, and data were presented following the post-infection challenge as no significant differences were observed following post-vaccination (*P ≤ 0.05 and **P ≤ 0.01). Orange bars refer to vaccinated dogs, and gray bars represent unvaccinated dogs.

**Fig 9:**
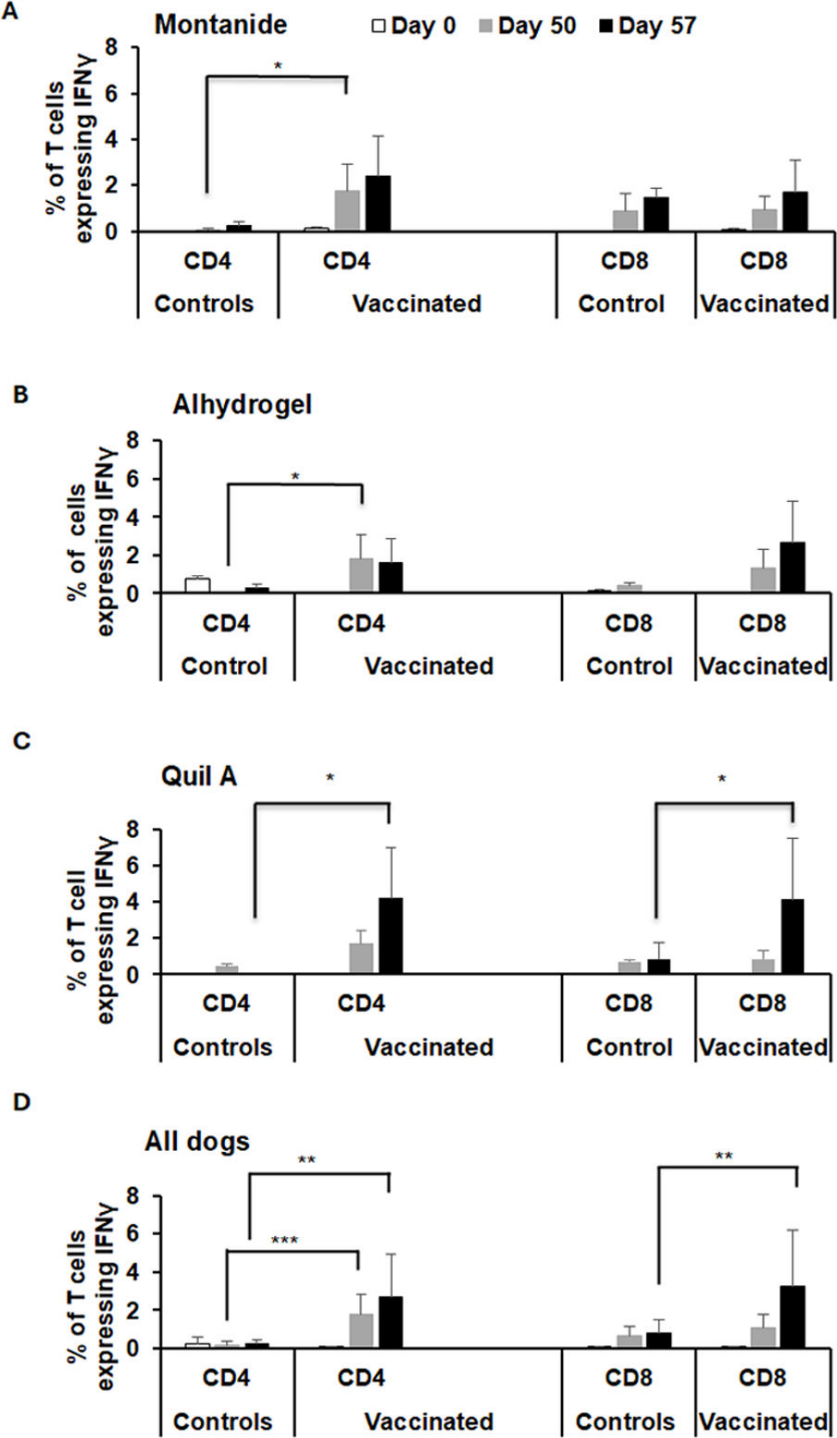
IFN-γ-expressing T cells are higher in vaccinated dogs. PBMCs recovered from all dogs were stimulated with *R. rickettsii* whole-cell antigens for 72 hours. Five to six hours prior to culture harvest, Brefeldin A was added to each culture well. Cells were then surface stained with antibodies specific to canine CD3, CD4, and CD8 antigens to capture all T cells and then checked for IFN-γ expression by flow cytometry analysis. The percentage of IFN-γ-expressing CD4 and CD8 T cells was measured. Both CD4 and CD8 T cells were significantly higher in vaccinated groups compared to unvaccinated infection controls. Panels **A–D** refer to the data generated for the Montanide group, Alhydrogel group, Quil A group, and for the data assessed for all three vaccinated groups, respectively. The vaccinated and control groups were compared by the two-way ANOVA test followed by multiplicity-adjusted *post hoc* comparisons (Tukey), and a *P*-value < 0.05 was considered statistically significant (*P ≤ 0.05, **P ≤ 0.01, and ***P ≤ 0.001).

Peripheral blood mononuclear cells (PBMCs) were collected from all dogs prior to vaccination (on day 0), prior to booster vaccination (day 30 post-primary vaccination), prior to infection challenge (day 50 post-vaccination, which is day 0 for infection challenge), and on days 7 and 14 post-infection challenge (which represent days 57 and 64 post-primary vaccinations). The cells were stimulated with whole-cell *R. rickettsii* antigens, and then the concentration of *R. rickettsii*-specific IFN-γ was measured in cell culture supernatants. There was no significant difference in IFN-γ production following primary vaccination between control and vaccinated dogs (not shown), but we observed a substantial increase in IFN-γ production by cells from vaccinated dogs at 20 days following booster vaccination (which is day 0 of infection challenge) relative to unvaccinated controls (Fig. 8). Furthermore, significant enhancement in IFN-γ production was also evident on day 7 post-infection in vaccinated dogs compared to unvaccinated controls. On day 14 post-infection, although cells from vaccinated dogs had a strong IFN-γ response, controls also generated a response; however, the difference was not statistically significant (Fig. 8). In parallel, we used intracellular cytokine staining to determine the frequency of *R. rickettsii-*specific IFN-γ-producing CD4^+^ T and CD8^+^ T cells in circulation on days 0, 50, and 57 post-primary vaccinations (note: day 57 is also day 7 post-infection challenge). The frequency of IFN-γ-producing T cells representing both cell types was greater in all vaccinated dogs (Fig. 9A through D). While an increased frequency of IFN-γ-positive CD4^+^ T cells was observed in dogs vaccinated with all three adjuvants, animals vaccinated with Quil A as the adjuvant had an increase in both CD4^+^ and CD8^+^ T cells producing IFN-γ (Fig. 9C).

### Vaccinated dogs developed minimal lesions

**Fig 10:**
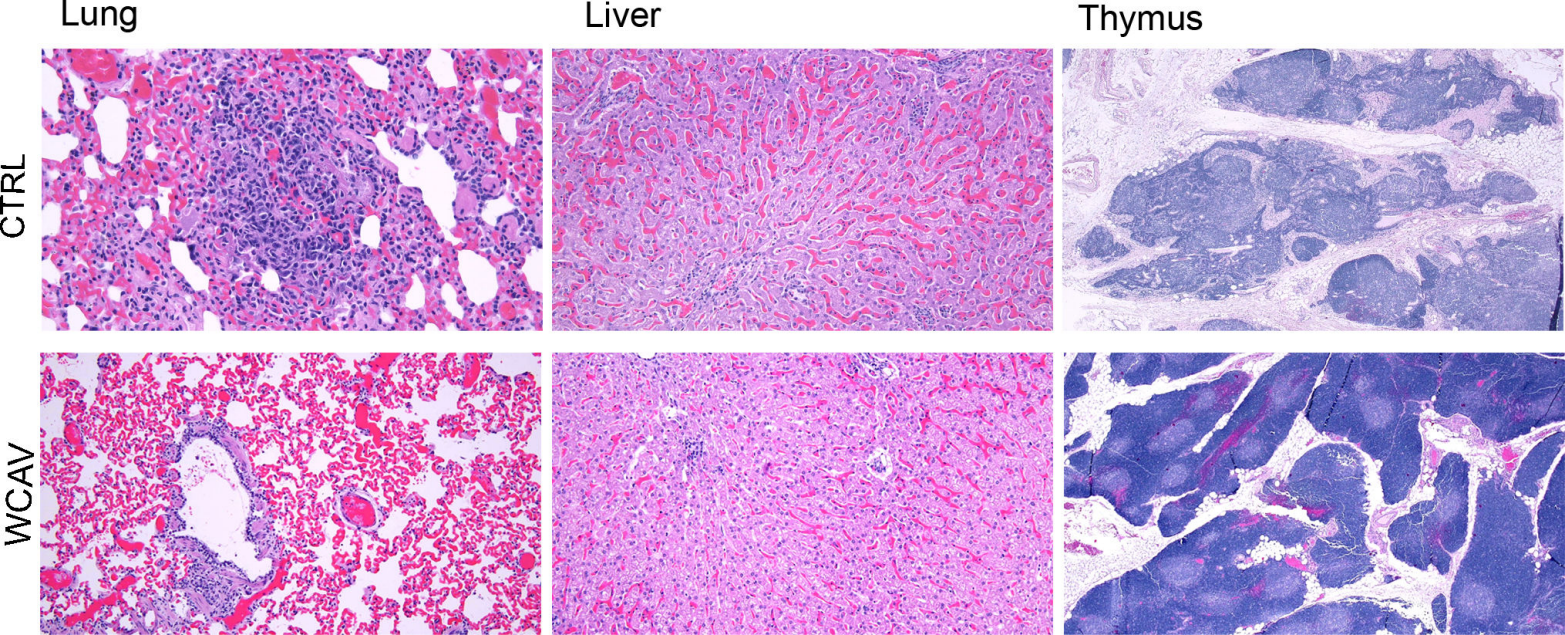
Histopathological observations in dogs impacted by vaccination. A randomly selected histology section from representative samples was shown with microscopic magnification for lung and liver at 100× magnification and thymus at 20×. Vaccinated dogs (WCAV) had multifocal minimal to mild inflammation in the lung and liver and no lesion in the thymus, while the unvaccinated group dogs (CTRL) had more severe inflammation in the lung and liver and diffuse moderate thymic atrophy.

Following the infection challenge, three of the six control dogs with clinical signs were euthanized on day 9 post-infection challenge. Three dogs of the vaccinated group were sacrificed to compare the differences in pathology in various tissue samples assessed. All three vaccinated dogs exhibited healthy tissue morphology, except for mild multifocal perivascular lymphocytic inflammation observed in the lungs (score 1) of two dogs, with one having mild multifocal hemorrhage in the brain (Table 5). The brain hemorrhage was not associated with any significant inflammation, and the cause remained uncertain. The other dog had mild multifocal lymphocytic inflammation in the liver (Fig. 10). Contrary to these observations, all three unvaccinated dogs had pronounced inflammatory scores (scores of 2 and 3) with lymphocytic and neutrophilic infiltration in the liver, spleen, heart, and lung tissues (Table 5; Fig. 10). Similarly, cortical lymphoid tissue atrophy and edema were seen in the thymus and tracheobronchial lymph node. All the remaining 12 vaccinated dogs and the three unvaccinated dogs assessed at the study end (~day 40 post-infection challenge) had relatively healthy tissue morphology except for minor disturbances that were independent of vaccine status (Table 6).

**TABLE 5:** Histopathological observations at day 9 post-infection^*a*^

Group	Adjuvant	Animal ID	Lung	Liver	Spleen	Heart	Thymus	Lymph node	Brain	Testis	Mammary gland
Vaccinated	Montanide	GJL2		M1L							X
	Quil A	FSK2	M1L							X	
	Alhydrogel	ZKL2	M1L						M1HE		X
Unvaccinated	Montanide	GIL2	M2LH	M1L	M2LH		D2AT				X
	Quil A	YBK2	M2LNH	M3L, EMH	M2LH	M1L	D3AT			X	
	Alhydrogel	IDL2	M3L, EMH	M2LN	M2L		D3AT	D1AT, D1ED		M1NL	X

^*a*^ M, multifocal; D, diffuse; inflammation score: 1, mild; 2, mild to moderate; 3, moderate; 4, moderate to severe; 5, severe; L, lymphocytic; N, neutrophilic; H, histiocytic; EMH, extramedullary hematopoiesis; AT, lymphoid atrophy; HE, hemorrhage; ED, edema; X, not present due to sex; and blank box, no lesion.

**TABLE 6:** Histopathological observations at the end of the study on days 37–41 post-infection^*a*^

Group	Adjuvant	Animal ID	Lung	Liver	Kidney	Spleen	Heart	Thymus	LN	Brain	Testis	Mammary gland	Bone marrow
Vaccinated	Montanide	GYL2	M2LH	M1L				D2AT				X	D2H
		YKK2		M1L							X		D2H
		FQK2	M1L								X		
		YGL2						D2AT				X	D2H
	Quil A	EOL2	M1LH									X	D2H
		XWK2		M1L	M2LP						X		D2H
		YTK2									X		
		IWL2										X	D2H
	Alhydrogel	XJK2	M2LH					D2AT		M1GLO, M1HE	X		D2H
		EGL2		M1L				D1AT				X	D2H
		IVL2	M1L									X	
		EJK2									X		
Unvaccinated	Montanide	EFK2									X		
	Quil A	GHL2	M1LH	M1L								X	D2H
	Alhydrogel	ZXK2	M1L	M1L							X		D2H

^*a*^ M, multifocal; D, diffuse; inflammation score: 1, mild; 2, mild to moderate; 3, moderate; 4, moderate to severe; 5, severe; L, lymphocytic; N, neutrophilic; H, histiocytic; P, plasmacytic; AT, lymphoid atrophy; HE, hemorrhage; GLO, gliosis; HYP, hyperplasia; X, not present due to sex; and blank box, no lesion.

The presence of *R. rickettsii* DNA was assessed in various tissue samples collected on day 9 or day 40 post-infection challenges. The tissues were analyzed by *adr2* nested PCR assay (Table 7). All tissues of the vaccinated dogs tested negative by the PCR assay independent of the adjuvants used (Table 7), whereas unvaccinated dogs had several tissue positives on day 9 post-infection challenge (Table 7). One of the three dogs from the unvaccinated group was also PCR positive in the lung and bone marrow on day 40 post-infection (Table 7).

**TABLE 7:** Infection status in tissue samples (Adr2 nested PCR assay)^*a*^

Group	Adjuvant	Animal	Lung	Liver	Spleen	Kidney	Thymus	Bone marrow	Cerebellum	Heart	Lymph node
Day 9 post-infection											
Controls	Montanide	GIL2	+	+	+	−	+	−	−	−	+
	Quil A	YBK2	+	+	+	−	−	−	−	−	+
	Alhydrogel	IDL2	+	+	+	+	+	+	+	−	+
Vaccinated	Montanide	GJL2	−	−	−	−	−	−	−	−	−
	Quil A	FSK2	−	−	−	−	−	−	−	−	−
	Alhydrogel	ZKK2	−	−	−	−	−	−	−	−	−
Days 37–41 post-infection											
Controls	Montanide	EFK2	+	−	−	−	−	+	−	−	−
	Quil A	GHL2	−	−	−	−	−	−	−	−	−
	Alhydrogel	ZXK2	−	−	−	−	−	−	−	−	−
Vaccinated	Montanide	GYL2	−	−	−	−	−	−	−	−	−
		YGL2	−	−	−	−	−	−	−	−	−
		FQK2	−	−	−	−	−	−	−	−	−
		YKK2	−	−	−	−	−	−	−	−	−
	Quil A	IWL2	−	−	−	−	−	−	−	−	−
		YTK2	−	−	−	−	−	−	−	−	
		EOL2	−	−	−	−	−	−	−	−	−
		XWK2	−	−	−	−	−	−	−	−	−
	Alhydrogel	XJK2	−	−	−	−	−	−	−	−	−
		EJK2	−	−	−	−	−	−	−	−	−
		EGL2	−	−	−	−	−	−	−	−	−
		IVL2	−	−	−	−	−	−	−	−	−

^*a*^ “+” indicates positive and “−” indicates negative.

## DISCUSSION

As *R. rickettsii* infection naturally occurs in dogs with similar clinical and pathological outcomes as in people, we previously developed the canine infection model that reproduced the fatal RMSF (43). We also described the assessment of two vaccine formulations in the canine model: a recombinant antigen vaccine (RCAV) containing two major immunogenic proteins and a whole cell inactivated antigen vaccine (WCAV), both of which are formulated with Montanide gel adjuvant (43). We discovered that WCAV offered complete protection against a virulent *R. rickettsii* infection challenge but not RCAV. Here, we tested the WCAV efficacy by comparing the effect of three commonly used adjuvants in the physiologically relevant canine infection model. We selected aluminum hydroxide-based adjuvant (Alhydrogel) and a saponin-based adjuvant (Quil A) and compared immune protection with the previously tested Montanide gel adjuvant. We demonstrated that WCAV offered similar vaccine protection as previously reported. The data independently confirmed our prior published work that WCAV offers sufficient immune protection to prevent the disease (43).

Adjuvants play a major role in inducing immunity, and immune responses can differ for different adjuvants (44). For example, Montanide-based vaccines aid in enhancing both humoral and cellular immune responses, inducing stronger Th1 responses without causing adjuvant-associated inflammatory disease in people and various animals, including in dogs (45–47). Alhydrogel is known to elicit stronger Th2 responses and higher IgG1 production compared to Montanide (48, 49). Saponin-based adjuvants, such as Quil A, in vaccine formulations are regarded as stimulating both Th1 and Th2 responses, as well as in enhancing cytotoxic T lymphocyte responses (50, 51).

Since *R. rickettsii* is an obligate intracellular bacterium that resides and replicates in the cytoplasm of vascular endothelial cells, it is important to define what type of immune stimulation is optimal for protection. Therefore, defining the contributions of adjuvants is a critical component to define vaccine efficacy. We performed the WCAV protection experiments using three different adjuvants: Montanide, Alhydrogel, and Quil A. Immune protection against the virulent *R. rickettsii* infection remained strong in all three adjuvant groups receiving the WCAV. Contrary to the protection provided by WCAV, unvaccinated dogs receiving the infection developed clinical signs and symptoms consistent with RMSF, including fever, petechial rashes, redness on the testis, and depression. Unvaccinated dogs also developed anemia, as indicated by the decline in hemoglobin in five of six dogs, while all 15 vaccinated dogs had normal hemoglobin levels regardless of the adjuvants used. Infection induced a significant drop in the total WBCs, which included a decline in the lymphocytes, neutrophils, and monocytes starting from day 10 post-infection challenge, as well as in causing persistent thrombocytopenia. WCAV prevented changes in WBC and platelet levels following infection challenge. The clinical signs observed in unvaccinated dogs with *R. rickettsii* infection were similar to those reported in several prior studies using the canine infection model (7, 43, 52, 53). Clinical signs, including rashes, anemia, and thrombocytopenia, are strong indicators of RMSF in human patients (14, 15). Independent of the adjuvant used, all vaccinated dogs had healthy physiology.

Systemic viable *R. rickettsii* was detected only in unvaccinated dogs that received the infection, while all vaccinated dogs cleared the infection from the blood, independent of the adjuvant. Infiltrating immune cells following an infection are activated by inflammatory cytokines such as IL-1, IL-6, and TNF-α, which are implicated in RMSF pathology (54, 55). Prior studies in human and canine RMSF patients report increases in IFN-γ, TNF-α, IL-6, IL-8, and MCP-1 during the acute phase of the disease (56–60). In our study, IFN-γ and IL-6 were the only cytokines significantly elevated post-infection, with a sharp increase observed in unvaccinated dogs on day 3. TNF-α levels differed between groups prior to vaccination and after infection challenge, and IL-8 and MCP-1 were elevated at isolated time points in vaccinated animals post-infection, though these changes were not sustained. Notably, although vaccinated animals did not show elevated systemic IFN-γ, they exhibited robust antigen-specific IFN-γ-producing T cell responses, highlighting the distinct nature of these measurements. Circulating IFN-γ captures broad inflammatory activity, potentially derived from multiple innate and adaptive cell types, whereas the PBMC assay measures antigen-specific T cell memory. Thus, the IFN-γ increase in unvaccinated dogs may reflect uncontrolled infection and generalized inflammation, while vaccinated dogs display effective T cell immunity without systemic cytokine escalation. Other cytokines showed individual variability and did not show a clear trend related to the effect of vaccination or infection. While IL-10 plays a complex role in modulating inflammation and pathogen clearance (61, 62), our data are insufficient to assign a specific functional role in this model. We, therefore, interpret these trends cautiously and do not infer a causal relationship between IL-10 levels and protection.

We also observed that ICAM-1, a vascular adhesion molecule associated with endothelial inflammation, was significantly elevated in unvaccinated dogs early after infection, with minimal expression in vaccinated animals. Although IL-6 is known to induce the expression of adhesion molecules such as ICAM-1, VCAM-1, and E-selectin (63, 64), the IL-6 levels in our study did not rise until after ICAM-1 expression had peaked, and two unvaccinated animals exhibited elevated baseline IL-6 at day 0, complicating interpretation of early cytokine dynamics. The timing of cytokine changes in our study suggests that other inflammatory mediators, such as IFN-γ, may have contributed to early ICAM-1 induction. Additionally, ICAM-1 can act as both an effector and amplifier of inflammation, engaging leukocyte β2-integrins and triggering downstream cytokine signaling (65, 66). These complex feedback pathways underscore the difficulty of establishing linear relationships between circulating cytokines and endothelial activation. Thus, while our cytokine and vascular marker data reveal a few statistically significant differences and several broader trends, we interpret these changes as descriptive features that align with observed differences in disease severity rather than mechanistic drivers of protection. Additional studies with larger cohorts and cellular-level resolution will be needed to clarify the immunological pathways involved in WCAV-mediated protection.

An infection-associated rise in antibody, primarily IgG2, was observed in the unvaccinated control dogs, while vaccinated dogs generated both IgG isotypes: IgG1 and IgG2. Notably, the increase in IgG2 in unvaccinated dogs occurred around 10–14 days post-infection, coinciding with when surviving animals began to resolve the disease. This suggests that protection depends more on the timely induction of antibody responses rather than on IgG2 alone being insufficient. Unvaccinated dogs were unable to contain the infection early, resulting in clinical disease, hematological and pathological changes, systemic infection, and elevated cytokine production. In contrast, vaccinated dogs contained infection before a strong inflammatory response developed, likely due to the earlier and broader IgG response coupled with T cell immunity. Canine IgG2 is associated with Th1-biased immune responses, which are important for controlling rickettsial infections (67).

Percentages of CD4^+^/IFNγ^+^ and CD8^+^/IFNγ^+^ T cells were significantly higher in all vaccinated groups, independent of the adjuvant used. Responses were highest in the Quil-A vaccinated group, consistent with the known ability of saponin-based adjuvants to induce strong cytotoxic CD8^+^ lymphocyte responses (49). The most significant difference was that Quil A had the highest IgG response and induced both Th1 and Th2 type antibody responses, although Montanide and Alhydrogel similarly triggered an *R. rickettsii* IgG2 response. We found no evidence that Quil A had an additional impact in offering immune protection as dogs from all three adjuvant groups were similarly resistant to the *R. rickettsii* challenge. However, given the greater magnitude of the T and B cell responses in the Quil A group, we speculate that it may offer more durable protection, although this hypothesis remains to be tested.

Pathological analysis of unvaccinated dogs that received infection presented infection-associated tissue lesions consistent with RMSF by day 9 after infection and were consistent with the RMSF-associated pathology reported earlier, such as granulomatous infiltration, atrophy, and edema (12, 13, 16, 43). We detected *R. rickettsii* DNA in various tissue samples from unvaccinated dogs, while all vaccinated dogs, independent of the adjuvants used, tested negative at both day 9 and day 40 after infection. Bacterial DNA was also absent in unvaccinated dogs when assessed on day 40 after infection, i.e., after the animals recovered from the initial phase of infection. Lesions were absent at later times in unvaccinated dogs, as well as in all vaccinated dogs irrespective of the adjuvants used, suggesting that unvaccinated animals also clear the infection-associated disease severity after several weeks, as suggested by the absence of clinically relevant lesions.

In conclusion, the results from the current study offer further evidence that the WCAV is an ideal candidate vaccine against RMSF, although additional investigations are necessary to determine if the vaccine can protect against the disease resulting from the bite of an infected tick. Similarly, additional investigations are warranted to define protection against heterologous strain infection challenges. Defining the value of vaccine formulations with three different adjuvants yielding sufficient immune protection is a major step toward furthering vaccine research progress in preventing RMSF disease in dogs and people. In particular, the WCAV is an ideal vaccine candidate that allows an option to choose more than one adjuvant for its use, although saponin-based adjuvants warrant further study for potential use in both companion animals and people.

## MATERIALS AND METHODS

### Propagation of *R. rickettsii* in Vero cells for the preparation of stocks

*R. rickettsii* (Sheila Smith strain) was grown in Vero (African green monkey kidney) cells (clone E6; ATCC CRL-1586) as previously described (43, 68, 69). Briefly, confluent monolayers of Vero cells grown in Dulbecco’s modified Eagle’s medium supplemented with 2% fetal bovine serum and 2 mM L-glutamine were infected with *R. rickettsii* at a multiplicity of infection of 1 and incubated in a 34°C incubator set at 5% CO_2_ until 50% of the monolayer was disrupted due to infection. Differential centrifugation of lysates from infected cells was performed to obtain the rickettsial stocks, which were resuspended in K-36 buffer (0.1 M potassium chloride, 0.015 M sodium chloride, and 0.05 M potassium phosphate buffer [pH 7.0]). The numbers of viable *R. rickettsii* organisms were determined by a plaque titration assay.

### Whole-cell antigen preparation for WCA vaccine

Antigens for WCA were prepared as described previously (43). Briefly, WCA was prepared from Vero cell cultures. The cultured organisms purified from the host cells were incubated in a 56°C water bath for 30 min, with mixing once every 10 min. The protein concentration was estimated according to the BCA (Bicinchoninic Acid) Protein Assay (ThermoFisher, Waltham, MA, USA).

### Vaccine formulations

Montanide pet gel (SEPPIC Inc., Fairfield, NJ, USA), Quil-A (InvivoGen, San Diego, CA, USA), and Alhydrogel (InvivoGen) were used as adjuvants in preparing the vaccines. Vaccines were prepared by mixing the equivalent of 70 µg/mL of inactivated *R. rickettsii* whole-cell antigens diluted in PBS with 2.5% Montanide pet gel or 250 µg of Quil A or 2% Alhydrogel.

### Experimental infections in dogs, health monitoring, and complete blood count analysis

Experiments with dogs complied with Public Health Service Policy on the Humane Care and Use of Laboratory Animals (70) and the U.S. Department of Agriculture (USDA) Animal Welfare Act and regulations (71). Purpose-bred beagles (11–12 months old of both sexes) were purchased from a class A USDA vendor (Ridglan Farms, Inc., Blue Mounds, WI, USA) and housed in indoor climate-controlled facilities at the University of Missouri. Dogs were provided with a commercially available dry dog food and water *ad libitum* and were also provided adequate space, allowing them to freely move about for regular exercise. Primary and booster vaccinations containing 70 µg of antigens each were performed on days 0 and 30, respectively. (The booster vaccine was stored at −20°C until use.) Each vaccine group consisted of five dogs (two or three each of males and females). Only adjuvants were administered to the control group animals (*n* = 6; two dogs each per adjuvant). All dogs were administered a vaccine or adjuvant by subcutaneous route. Twenty days following the booster vaccination (day 50), all dogs were challenged intravenously with *R. rickettsii* (~10^5^ organisms) per dog in a 1 mL volume of PBS. All dogs received diphenhydramine syrup (4 mg/kg of body weight) orally about 30 min before the infection challenge to avoid any possible anaphylactic shock (72). All dogs from the four groups—three vaccination groups and the unvaccinated group (*n* = 21)—were monitored daily for health, clinical, and behavioral changes and twice weekly for hematological variations by CBC analysis using the ProCyte DX (IDEXX, Westbrook, ME, USA). Body temperatures were measured twice a week during the vaccination phase and daily following infection challenges; temperature measures were taken at similar times each day mostly between 9 and 10 a.m. Blood samples (2 mL) were collected from the cephalic vein into EDTA tubes once every 2 days from day 0, and the blood collections continued until the study end. Blood samples of 4 mL each were also collected in sodium citrate CPT (BD Biosciences, San Jose, CA, USA) tubes for peripheral blood mononuclear cells stimulation to measure the T cell proliferation. At the end of the study (~day 40 post-infection challenge), all animals were euthanized in accordance with the recommendations of the Panel on Euthanasia of the American Veterinary Medical Association using a commercial euthanasia solution (Fatal-Plus solution at 0.22 mL/kg [86 mg/kg of pentobarbital]). Three dogs, each from the unvaccinated and vaccinated group (one each from a different adjuvant-vaccinated group), were also euthanized at day 9 post-infection challenge during the acute presentation of the disease.

### Detection of *R. rickettsii* by culture recovery

Blood samples in EDTA recovered on days 7, 14, and 21 post-infection challenge were used for the culture recovery experiment. Plasma fractions were removed from 4 mL of whole blood, and the buffy coats were carefully transferred into a 15 mL sterile Falcon centrifuge tube containing 5 mL red blood cell lysis buffer (155 mM NH_4_Cl, 10 mM KHCO_3_, and 0.1 mM EDTA) and mixed several times until complete RBC lysis occurred. The samples were then centrifuged at 300 × *g* for 5 min, and the supernatants were discarded. Buffy coat pellet from each sample was resuspended in 200 µL of sterile 1× PBS. One hundred microliters each of the cell suspension was transferred to a well in a 12-well sterile culture plate containing a monolayer of Vero cells (about 80% confluent). The culture plates were transferred to a CO_2_ incubator at 37°C, and growth of *R. rickettsii* was assessed as per the detailed culture protocols described above. The presence of *R. rickettsii* infection in the cultured Vero cells was determined following the culture being transferred onto a slide, which was then fixed and stained with Hema3 staining protocol as in reference 72. The presence of *R. rickettsii* was assessed in culture by this method by monitoring the cultures for up to 34 days.

### Rickettsial DNA assessed by nested PCR assay

A hundred microliters of blood samples collected in EDTA tubes and 25 mg each of various tissue samples and 10 mg of spleen sample were used to recover total genomic DNA using a DNeasy Blood and Tissue Kit (Qiagen, Valencia, CA, USA) according to the manufacturer’s protocol. Final purified DNA from blood and/or tissue was stored at −20°C until used in the nested PCR assay targeting the *adr2* gene of *R. rickettsii*, following the method described previously (43).

### Quantification of cytokines and ICAM-1

Plasma cytokine (IL-2, TNF-α, IL-6, IFNγ, IL-12/IL-23p40, IL-8, MCP-1, and IL-10) levels were assessed using Luminex technology and the Cytokine/Chemokine/Growth Factor 11-Plex Canine ProcartaPlex Panel 1 kit (ThermoFisher Scientific, USA). The analyses were performed according to the instructions from the manufacturer. Plasma levels of ICAM-1 were analyzed for different time points by using commercial ELISA kits (Cusabio Dog intercellular adhesion molecule 1, ICAM-1/CD54 ELISA Kit).

### Humoral immune response

Enzyme-linked immunosorbent assays were performed using *R. rickettsii* inactivated whole-cell antigens to measure the IgG expression. Briefly, plasma samples from peripheral blood samples collected in EDTA tubes were obtained by standard procedures. Ninety-six well Immulon 2HB microtiter plates (Thermo Fisher Scientific, Waltham, MA, USA) were coated overnight at 4°C with 100 µL of purified *R. rickettsii* antigens (2 µg/mL) in the coating buffer (Na2CO_3_-NaHCO_3_ buffer, pH 9.6). Subsequently, the plates were blocked with 200 µL of the blocking buffer (1% BSA in 1× PBS) for 1 hour at 37°C. Plasma samples for each time point were diluted 1:200 in the blocking buffer; 100 µL of each sample was transferred per well, and all samples were assessed in triplicate. The plates were incubated for 1 hour at 37°C and then washed three times in 200 µL each of wash buffer (1× PBS containing 0.05% Tween 20) per well. One hundred microliters of a 1:50,000 dilution of anti-canine total IgG (PA1-29738, ThermoFisher, USA), 1:10,000 anti IgG1 (AHP947P, Bio-Rad, Hercules, CA, USA), or 1:5,000 of anti IgG2 (AHP948P, Bio-Rad, Hercules, CA, USA) peroxidase-labeled antibodies was added to each well, and plates were incubated for 45 min at 37°C. Subsequently, plates were washed three times with 200 µL of wash buffer per well. Finally, 100 µL of TMB substrate was added, and the reaction was allowed to develop the color for up to 10 min. The reaction was stopped by adding 50 µL of 1 M phosphoric acid, and absorbance was measured at 450 nm using an ELISA plate reader (Tecan-infinite M nano, Männedorf, Switzerland).

### Isolation of PBMCs and assessment of T cell expansion and cytokine analysis following *R. rickettsii* antigen stimulation

This experiment was performed as previously described (73). Briefly, about 8–10 mL of blood was collected from each animal in sodium citrate CPT tubes (BD Biosciences, San Jose, CA, USA) at the indicated time points after WCAV vaccination and following infection challenge with the virulent Sheila Smith strain of *R. rickettsii*. The blood tubes were centrifuged as per the manufacturer’s instructions prior to shipping overnight to Iowa State University, where the analysis was performed. The harvested PBMCs were washed twice with complete RPMI media; cells were counted and adjusted to 4 × 10^6^ PBMCs/mL.

### ELISA for canine IFN-γ

PBMCs were collected on different days following vaccination and *R. rickettsii* infection challenge. Cells were isolated by density centrifugation from buffy coat fractions of peripheral blood collected in acid citrate dextrose. Cells were washed and resuspended in complete RPMI 1640 (Gibco, Carlsbad, CA, USA) supplemented with 2 mM L-glutamine, 25 mM HEPES buffer, a 1% antibiotic-antimycotic solution, 50 mg/mL gentamicin sulfate, 1% nonessential amino acids, 2% essential amino acids, 1% sodium pyruvate, 50 M 2-mercaptoethanol, and 10% (vol/vol) fetal bovine serum. Cells were cultured at 37°C with 4 × 10^5^ cells/well in 96-well plates and stimulated with 10 µg/mL *R*. *rickettsii* whole-cell antigens. As a positive control, cells were stimulated with 5 µg/mL of Concanavalin A (Sigma-Aldrich). Negative-control wells remained unstimulated. PBMC culture supernatants were collected after 5 days of stimulation, and the presence of canine IFN-γ in the supernatants was assessed by using a commercial ELISA kit (R&D Systems, Minneapolis, MN, USA) according to the manufacturer’s instructions.

### IFN-γ intracellular staining and flow cytometry

Briefly, PBMCs were stimulated with *R. rickettsii* whole-cell antigens and were then cultured with Brefeldin A during the final 5–6 hours of incubation. Following stimulation, cells were surface stained with mouse anti-canine CD3-FITC (clone CA17.2112), CD4-PECy7 (clone YKIX302.9), and CD8- and mouse-anti-bovine IFNγ-APC (clone CC302), obtained from BioRad Laboratories (Hercules, CA, USA). PBMCs were resuspended in FACS buffer (0.1% NaN3, 10% fetal calf serum, and PBS) and incubated with primary antibodies at 10 µg/mL for 20 min at 4°C. Cells were then fixed using BD FACS Lysis buffer (BD Biosciences, San Jose, CA, USA). Cytokine intracellular staining for IFNγ was performed using the BD Fixation and Permeabilization Solution Kit (BD Biosciences, San Jose, CA, USA). Flow cytometry data were acquired on a BD FACSCanto flow cytometer and analyzed with FlowJo software Version 10.10 (BD Biosciences, San Jose, CA, USA).

### Histopathology analysis

The sample collections for the gross and histopathological analysis were carried out by a board-certified veterinary pathologist (D. Y. Kim) with no knowledge about the vaccination status (blind study). We opted for this approach to avoid any bias during histological evaluation of the samples. Selected tissues from all dogs, including lung, liver, thymus, kidney, spleen, heart, lymph nodes, bone marrow, testicle or mammary glands, and brain, were fixed in 10% neutral buffered formalin and processed to generate 4 µm sections and stained with hematoxylin and eosin. A comprehensive numeric score was used to subjectively grade the severity of the lesions, including inflammation in all organs examined: 1, mild; 2, mild to moderate; 3, moderate; 4, moderate to severe; and 5, severe. The distribution of the lesions was divided into multifocal or diffuse. The overall predominant cell types (lymphocytic, neutrophilic, plasmocytic, and histiocytic) were assessed in the area of inflammation in the examined organs.

### Statistical analysis

Statistical analysis in GraphPad Prism 10 was performed by a two-way ANOVA test with Tukey’s *post hoc* test to analyze differences between controls and vaccinated dogs and among the three adjuvant groups of vaccinated dogs.

## SUPPLEMENTAL MATERIAL

Supplemental material iai.00412-25-s0001.pptx Fig. S1 to S4.

ASM does not own the copyrights to Supplemental Material that may be linked to, or accessed through, an article. The authors have granted ASM a non-exclusive, world-wide license to publish the Supplemental Material files. Please contact the corresponding author directly for reuse.
