# Supplementary material for: Rickettsia rickettsii inactivated whole cell antigen vaccine protects against Rocky Mountain spotted fever independent of the adjuvant used
Source: Infect Immun. 2025 Oct 29;93(12):e00412-25. doi: 10.1128/iai.00412-25 (PMC12707144; doi:10.1128/iai.00412-25)
Supplement: Supplemental material — Fig. S1 to S4. [file iai.00412-25-s0001.pptx]

## Slide 1
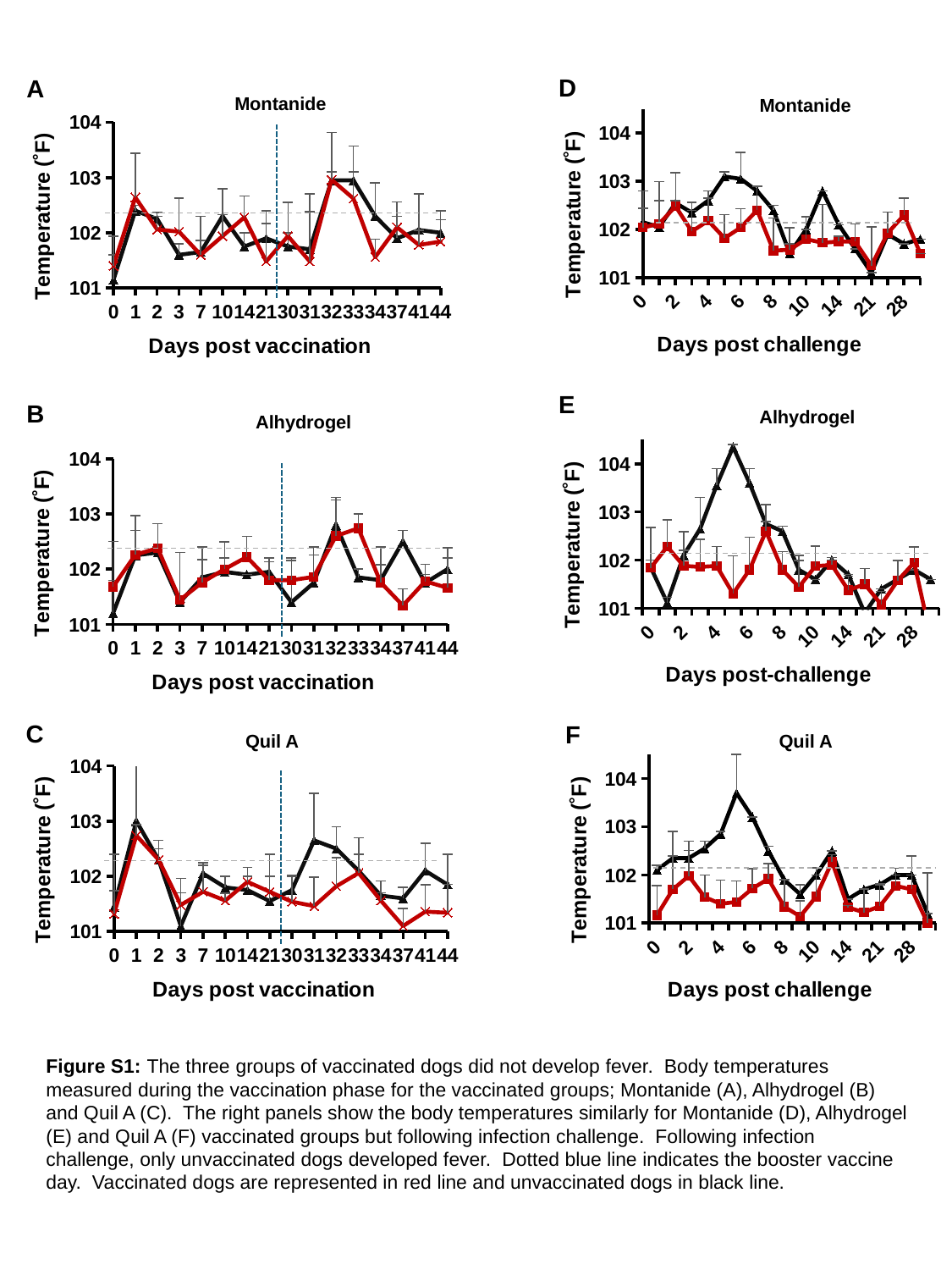

D
A
Montanide
Montanide
### Chart
| Category | Montanide Controls | Montanide Vaccinated |
|---|---|---|
| 0 | 102.15 | 102.04 |
| 1 | 102.05 | 102.11999999999999 |
| 2 | 102.55 | 102.47999999999999 |
| 3 | 102.35 | 101.96000000000001 |
| 4 | 102.6 | 102.17999999999999 |
| 5 | 103.1 | 101.82 |
| 6 | 103.05 | 102.03999999999999 |
| 7 | 102.80000000000001 | 102.4 |
| 8 | 102.4 | 101.56 |
| 9 | 101.5 | 101.58 |
| 10 | 102.0 | 101.8 |
| 12 | 102.8 | 101.725 |
| 14 | 102.1 | 101.75 |
| 16 | 101.6 | 101.75 |
| 21 | 101.1 | 101.25 |
| 23 | 101.9 | 101.925 |
| 28 | 101.7 | 102.3 |
| 37 | 101.8 | 101.5 |
### Chart
| Category | Montanide Controls | Montanide Vaccinated |
|---|---|---|
| 0 | 101.15 | 101.39999999999999 |
| 1 | 102.4 | 102.64000000000001 |
| 2 | 102.25 | 102.06000000000002 |
| 3 | 101.6 | 102.02000000000001 |
| 7 | 101.65 | 101.6 |
| 10 | 102.3 | 101.94 |
| 14 | 101.75 | 102.28 |
| 21 | 101.9 | 101.47999999999999 |
| 30 | 101.75 | 101.94000000000001 |
| 31 | 101.7 | 101.47999999999999 |
| 32 | 102.94999999999999 | 102.96 |
| 33 | 102.94999999999999 | 102.62 |
| 34 | 102.30000000000001 | 101.55999999999999 |
| 37 | 101.9 | 102.10000000000001 |
| 41 | 102.05000000000001 | 101.78 |
| 44 | 102.0 | 101.83999999999999 |E
B
Alhydrogel
Alhydrogel
### Chart
| Category | Alhydrogel- Control | Alhydrogel-Vaccinated |
|---|---|---|
| 0 | 101.85 | 101.84 |
| 1 | 101.11500000000001 | 102.28 |
| 2 | 102.1 | 101.88 |
| 3 | 102.65 | 101.86 |
| 4 | 103.55000000000001 | 101.88000000000001 |
| 5 | 104.35 | 101.3 |
| 6 | 103.6 | 101.8 |
| 7 | 102.75 | 102.6 |
| 8 | 102.6 | 101.8 |
| 9 | 101.8 | 101.44 |
| 10 | 101.6 | 101.87500000000001 |
| 12 | 102.0 | 101.89999999999999 |
| 14 | 101.7 | 101.375 |
| 16 | 100.9 | 101.5 |
| 21 | 101.4 | 101.075 |
| 23 | 101.6 | 101.57499999999999 |
| 28 | 101.8 | 101.94999999999999 |
| 37 | 101.6 | 100.4 |
### Chart
| Category | Alhydrogel- Controls | Alhydrogel-Vaccinated |
|---|---|---|
| 0 | 101.19999999999999 | 101.67999999999999 |
| 1 | 102.25 | 102.26 |
| 2 | 102.30000000000001 | 102.38000000000001 |
| 3 | 101.4 | 101.44 |
| 7 | 101.85 | 101.75999999999999 |
| 10 | 101.95 | 102.0 |
| 14 | 101.9 | 102.22 |
| 21 | 101.95 | 101.8 |
| 30 | 101.4 | 101.8 |
| 31 | 101.75 | 101.85999999999999 |
| 32 | 102.8 | 102.6 |
| 33 | 101.85 | 102.74000000000001 |
| 34 | 101.80000000000001 | 101.76000000000002 |
| 37 | 102.5 | 101.34 |
| 41 | 101.75 | 101.78 |
| 44 | 102.0 | 101.66 |C
F
Quil A
Quil A
### Chart
| Category | QuilA - Controls | QuilA Vaccinated |
|---|---|---|
| 0 | 101.45 | 101.31999999999998 |
| 1 | 103.0 | 102.74000000000001 |
| 2 | 102.3 | 102.3 |
| 3 | 101.1 | 101.48 |
| 7 | 102.05000000000001 | 101.72 |
| 10 | 101.8 | 101.56000000000002 |
| 14 | 101.75 | 101.9 |
| 21 | 101.55 | 101.72 |
| 30 | 101.75 | 101.53999999999999 |
| 31 | 102.65 | 101.46000000000001 |
| 32 | 102.5 | 101.82000000000001 |
| 33 | 102.1 | 102.06 |
| 34 | 101.65 | 101.56 |
| 37 | 101.6 | 101.10000000000001 |
| 41 | 102.1 | 101.36 |
| 44 | 101.85 | 101.34 |
### Chart
| Category | QuilA - Controls | QuilA Vaccinated |
|---|---|---|
| 0 | 102.1 | 101.16 |
| 1 | 102.35 | 101.7 |
| 2 | 102.35 | 101.97999999999999 |
| 3 | 102.55000000000001 | 101.54 |
| 4 | 102.85 | 101.4 |
| 5 | 103.7 | 101.44 |
| 6 | 103.2 | 101.72 |
| 7 | 102.5 | 101.92 |
| 8 | 101.9 | 101.33999999999999 |
| 9 | 101.6 | 101.14 |
| 10 | 102.0 | 101.55 |
| 12 | 102.5 | 102.275 |
| 14 | 101.5 | 101.32499999999999 |
| 16 | 101.7 | 101.225 |
| 21 | 101.8 | 101.35000000000001 |
| 23 | 102.0 | 101.775 |
| 28 | 102.0 | 101.7 |
| 37 | 101.2 | 101.0 |Figure S1: The three groups of vaccinated dogs did not develop fever. Body temperatures measured during the vaccination phase for the vaccinated groups; Montanide (A), Alhydrogel (B) and Quil A (C). The right panels show the body temperatures similarly for Montanide (D), Alhydrogel (E) and Quil A (F) vaccinated groups but following infection challenge. Following infection challenge, only unvaccinated dogs developed fever. Dotted blue line indicates the booster vaccine day. Vaccinated dogs are represented in red line and unvaccinated dogs in black line.

## Slide 2
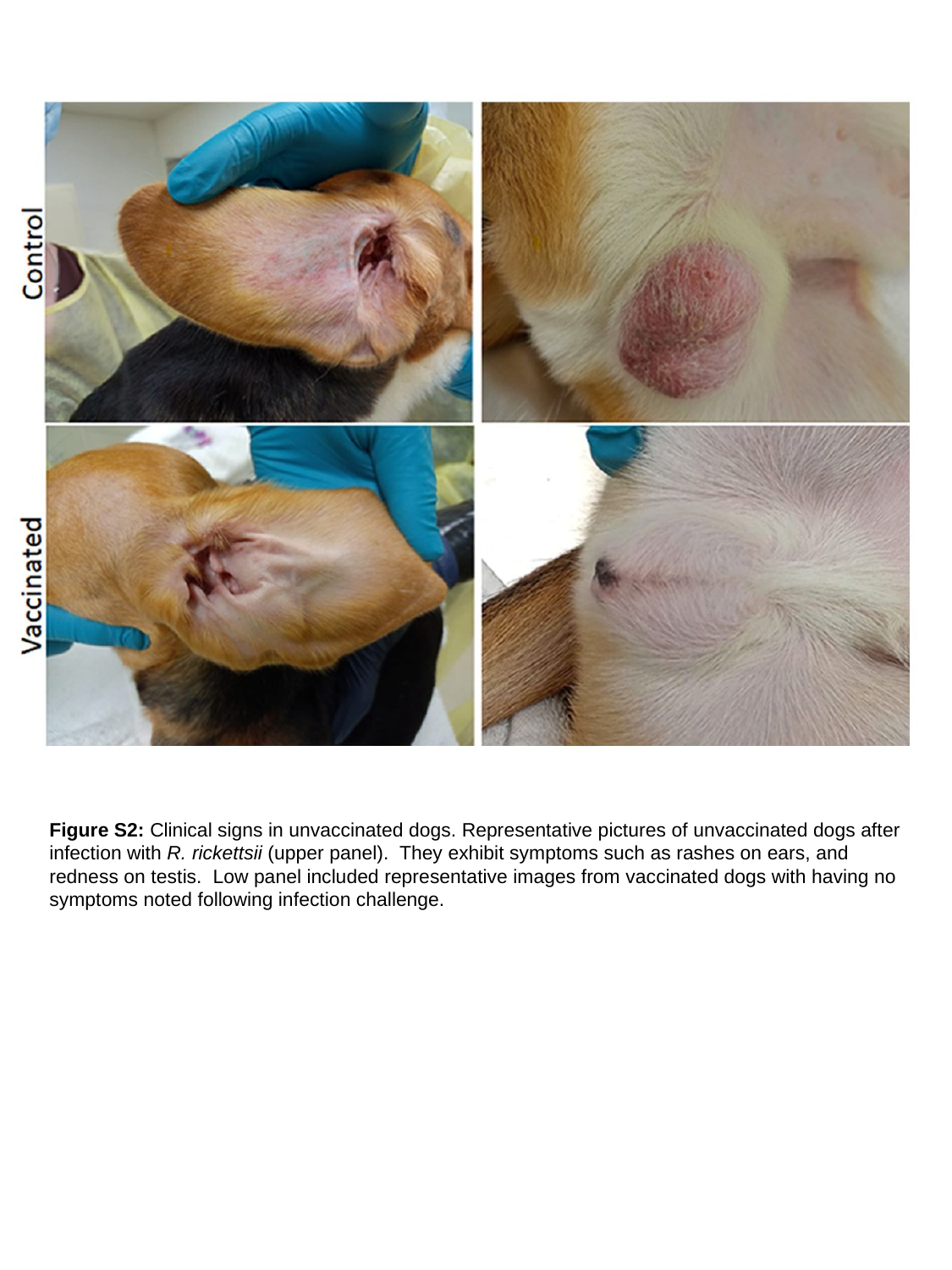

Figure S2: Clinical signs in unvaccinated dogs. Representative pictures of unvaccinated dogs after infection with R. rickettsii (upper panel). They exhibit symptoms such as rashes on ears, and redness on testis. Low panel included representative images from vaccinated dogs with having no symptoms noted following infection challenge.

## Slide 3
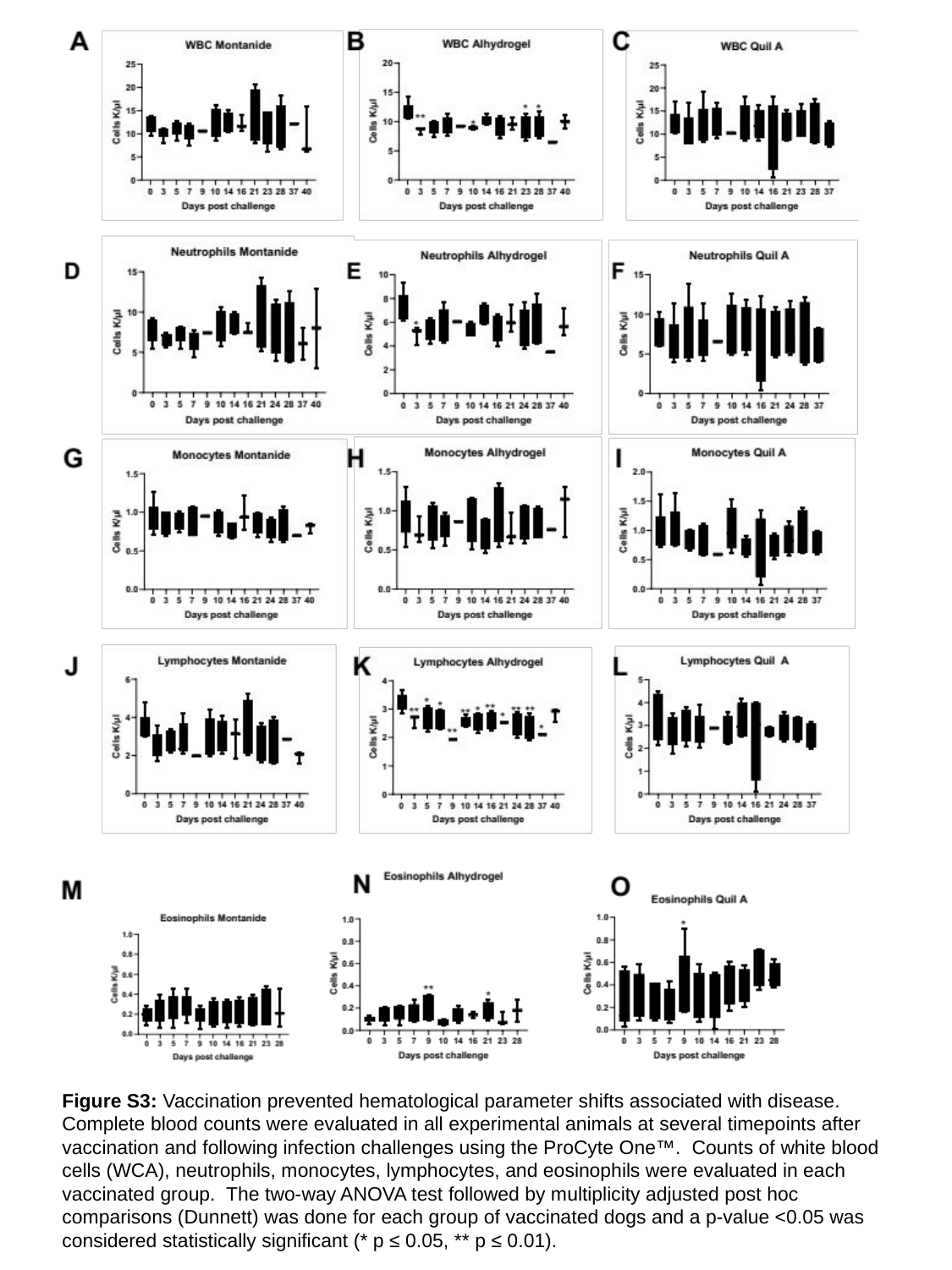

Figure S3: Vaccination prevented hematological parameter shifts associated with disease. Complete blood counts were evaluated in all experimental animals at several timepoints after vaccination and following infection challenges using the ProCyte One™. Counts of white blood cells (WCA), neutrophils, monocytes, lymphocytes, and eosinophils were evaluated in each vaccinated group. The two-way ANOVA test followed by multiplicity adjusted post hoc comparisons (Dunnett) was done for each group of vaccinated dogs and a p-value <0.05 was considered statistically significant (* p ≤ 0.05, ** p ≤ 0.01).

## Slide 4
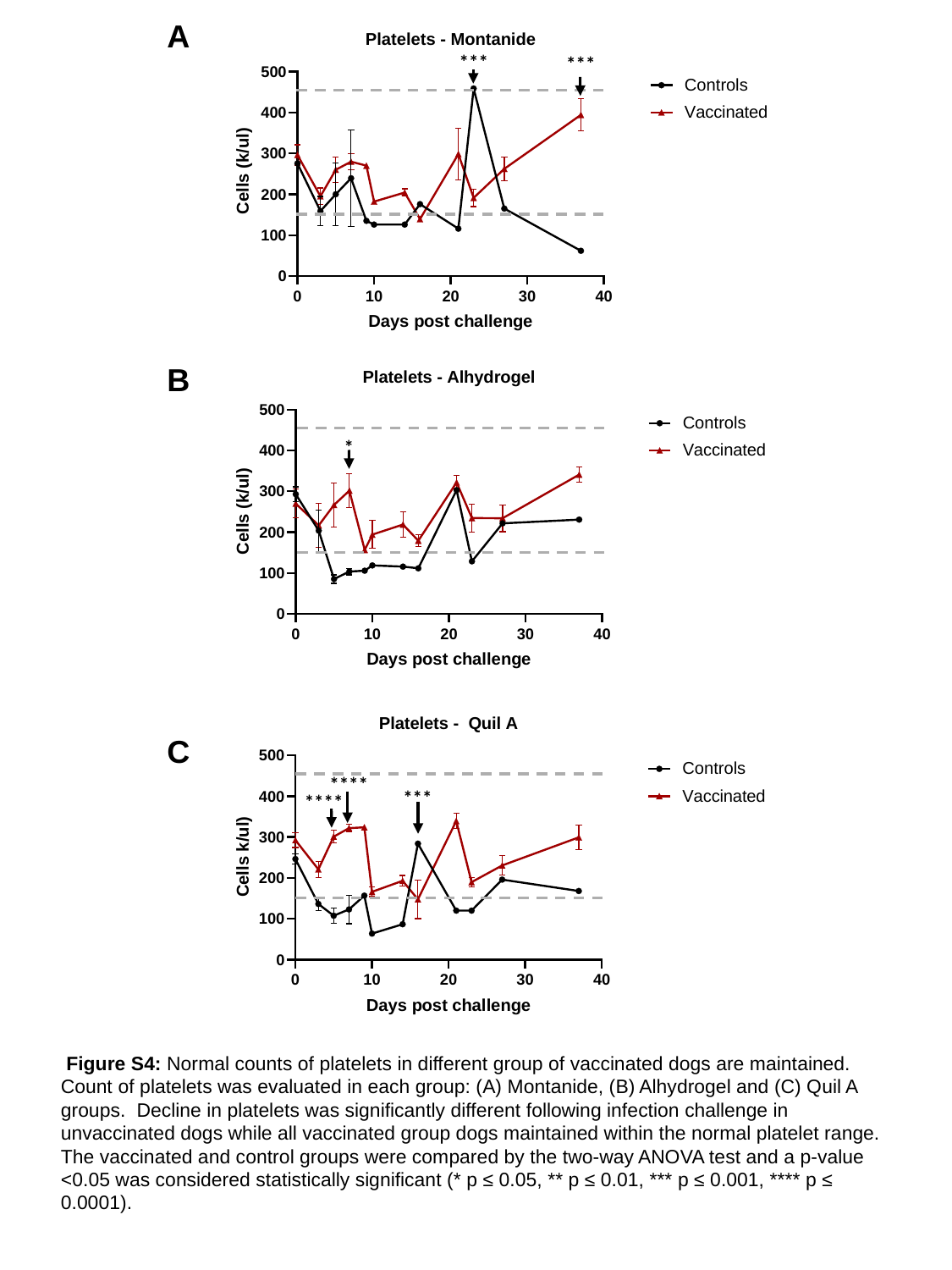

A
***
***
B
*
****
***
****
C
 Figure S4: Normal counts of platelets in different group of vaccinated dogs are maintained. Count of platelets was evaluated in each group: (A) Montanide, (B) Alhydrogel and (C) Quil A groups. Decline in platelets was significantly different following infection challenge in unvaccinated dogs while all vaccinated group dogs maintained within the normal platelet range. The vaccinated and control groups were compared by the two-way ANOVA test and a p-value <0.05 was considered statistically significant (* p ≤ 0.05, ** p ≤ 0.01, *** p ≤ 0.001, **** p ≤ 0.0001).
